# Differentially expressed genes in a flock of Chinese local-breed chickens infected with a subgroup J avian leukosis virus using suppression subtractive hybridization

**DOI:** 10.1590/S1415-47572009005000097

**Published:** 2010-03-01

**Authors:** Guiping Zhao, Maiqing Zheng, Jilan Chen, Jie Wen, Chunmei Wu, Wenjuan Li, Libo Liu, Yuan Zhang

**Affiliations:** 1 Institute of Animal Sciences, Chinese Academy of Agricultural Sciences, BeijingChina; 2 Department of Animal Genetics Breeding, China Agricultural University, BeijingChina

**Keywords:** Avian leukosis virus subgroup J, chicken, transferring gene, MHC genes, hemoglobin gene

## Abstract

Avian leukosis virus subgroup J (ALV-J) is a new type of virus that mainly induces myeloid leukosis (ML) in chickens. To further elucidate the pathogenesis of ALV-J infection and tumor development, expression profiles from the bone marrow tissue of 15 infected and 18 non-infected birds from a local-breed poultry-farm under naturally infected conditions, were analyzed by suppression-subtractive hybridization. The birds were diagnosed as ML+ (or ML-) by specific ALV-J detection methods, involving serological tests for antigens and antibodies, and RT-PCR to detect viral RNA. A total of 59 partial gene sequences were revealed by differential screening of 496 forward and 384 reverse subtracted cDNA clones. Of these, 22 identified genes, including 8 up-regulated and 14 down-regulated, were related to immune functions, these genes being, MHC B-G antigen, translationally-controlled tumor protein (TPT1/TPTC), transferrin and ferritin, hemoglobin and Carbonic anhydrase. Four of the down-regulated genes were selected for further analysis, in view of their predicted roles in infection and immunity by real-time qRT-PCR, using RNA collected from the same birds as those used for SSH. The four genes were expressed at significantly lower levels (p < 0.001) in ALV-J infected birds than in non-infected ones.

## Introduction

Avian leukosis viruses (ALV) constitute a group of avian retroviruses that induce malignant neoplasm in poultry. There are six well-characterized chicken subgroups of ALV (A to E and J) identified by the envelope glycoprotein responsible for specific viral interference patterns, host-range and virus neutralization (Payne *et al.*, 1992). The most recently identified subgroup, ALV-J, first isolated in the UK from broiler chickens in 1988 (Payne *et al.*, 1991), was found to be associated with myeloid leukosis (ML). Although initially it was assumed that the virus was restricted to the UK, cases of ML in broiler breeding-flocks have cropped up in several other European countries (Payne, 1998), Africa (Aly, 2000) and Australia (Bagust *et al.*, 2004), thus indicating its world-wide distribution among commercial meat-type poultry (Landman *et al.*, 2002).

In China, it has been shown that ML caused by ALV-J was to be found in both meat-type (Cui *et al.*, 2002) and egg-type (Xu *et al.*, 2004) chickens nation-wide. Moreover, recent outbreaks have occurred in local breeding flocks (Cheng *et al.*, 2005; Sun and Cui, 2007). Mortality varied from 5% to 20% in flocks of various breeds, thereby causing severe damage to the domestic chicken industry (Cui *et al.*, 2002; Sun and Cui, 2007).

Whereas receptor genes of subgroups A, B, C, D and J were identified by using transfected cultured cell methods (Bates *et al.*, 1993; Young *et al.*, 1993; Barnard and Young, 2003; Elleder *et al.*, 2005; Chai and Bates., 2006), in ALV infection and tumor metastasis, the leading causes of tumor-related deaths, this involves making use of multiple genes and their products. Little is known regarding either the mechanism underlying pathogenesis in avian leukosis in the field or the changes in gene expression during processes of infection and tumor development. The elucidation of gene expression in specific target tissues with different potentials for metastasis might lead to understanding the molecular mechanisms of infection and metastasis (Edgar *et al.*, 2006).

For the present study, a flock was spontaneously infected with ALV-J, whereupon PCR-based suppressive subtractive hybridization (SSH) methods were applied to identify genes that were up and down-regulated. This study will aid in discovering candidate genes and thus contribute to a better understanding of the disease itself.

## Material and Methods

###  Birds

The chickens used in this study came from a locally raised pure-line Chinese breed, with a past history (2004) of high morbidity in 22 to 35 week-old birds, mortality reaching 15 to18% in a flock of about 2000 birds. According to the attending vet., an initial postmortem examination of sick and dead birds revealed the cause to be ML+, Furthermore, there appeared characteristic pale-white lesions on the inner surface of the sternum, an enlarged spleen and occasional gray-white hepatic nodules.

The diagnosis of ALV-J infection was with serological tests for antigens and antibodies, and reverse transcription polymerase chain reaction (RT-PCR) for detecting type-specific viral RNA. 148 female chickens, 15 sick and 133 healthy birds (health-status was defined solely from appearance), were chosen when 33 weeks old. Antibodies against ALV subgroup J were detected by enzyme-linked immunosorbent assay (ELISA) (IDEXX Laboratories). The presence of the p27 antigen was tested on serum and cloacal swabs, using the antigen capture ELISA kit from the same supplier. H5 and H7 primer sets were used for RT-PCR amplification of the 3' and 5'ends of the ALV-J *env* viral genes for analysing genomic RNA isolated from bone marrow and the spleen (Smith *et al.*, 1998; Stedman and Brown, 1999). All test results were positive for the 15 overtly sick chickens, with the isolated ALV-J revealing high identity (99%) for gp85 to strain NX0101 (an ALV-J strain isolated in northern China (GenBank accession number AY897227.1, http://www.ncbi.nlm.nih.gov/nuccore/60300037; data not shown). These birds were considered to be congenitally infected with ALV-J and were denominated Group A.

Among the 133 apparently healthy birds, only 18 were negative in all the tests. Thus, these were considered as free of infection and designated Group B.

###  Subtracted cDNA library construction

Bone marrow tissue collected from these two groups of birds was used for SSH. RNA was extracted directly from the marrow using a Qiagen kit, and two RNA pools (A and B) were constructed. Poly (A+) mRNA from each pool was purified using the PolyATtract mRNA Isolation System (Promega Corp).

Suppression subtractive hybridization was performed using the PCR-Select cDNA Subtraction Kit (Clontech), according to manufacturer's instructions. For the Forward SSH library, Poly (A+) mRNA from infected chickens (A pool, n = 15) was used as the “tester”, or the population whose up-regulated transcripts were to be identified and Poly (A+) mRNA from the B pool (n = 18) served as the “driver” or reference population of transcripts. A Reverse SSH library was also constructed using pools A and B in reverse fashion. The recommended controls were included and the quality of the subtraction was evaluated by PCR using primers specific to chicken G3PDH mRNA and control tissues.

Differentially expressed PCR products were cloned using a pMD18-T plasmid vector (TaKaRa) and DH5α cells by standard procedures. Recombinant white colonies were randomly selected and expanded in LB broth containing ampicillin, followed by plasmid extraction.

###  Reverse Northern blotting

Subtracted clones were subjected to differential screening to confirm their unique gene expression. Reverse Northern blotting was performed using the DIG High Prime DNA Labeling and Detection Starter Kit I (Roche, Genmany). ^32^P-dCTP-labelled probes were synthesized using cDNA produced from the same pools (A and B) of poly(A)+ mRNA used for SSH.

Briefly, the cDNA inserts in plasmids were amplified by PCR in a total volume of 25 μL, under the following conditions: 94 °C for 5 min. followed by 35 cycles at 94 °C for 30 s, 68 °C for 30 s, 72 °C for 45 s and a final cycle at 72 °C for 10 min. PCR products were analyzed on 2% agarose gels to identify inserts containing clones. Each PCR product from a positive colony was denatured at 94 °C for 10 min and 1 μL was dot-blotted on to a Hybond-N nylon membrane (Amersham Pharmacia Biotech). After hybridization and washes, membranes were exposed to X-ray film (Kodak, GE Healthcare Europe). Images were acquired using the UVP BioImaging system (EC3 system, USA), and analyzed with Quantity One quantification software. cDNA clones were considered to be differentially expressed when blots probed with the subtracted tester repeatedly demonstrated a signal intensity > 2-fold different than blots probed with the subtracted driver.

###  Sequencing positive clones

Plasmids from a total of 64 forward and 40 reverse subtracted clones representing differentially expressed genes were sequenced by dideoxy chain termination using an ABI 3700 DNA Analyzer (Shanghai Sangon Biological Engineering Technology Co., Ltd). Sequences were compared with the GenBank database using a BLASTN search.

###  Real-time PCR (qRT-PCR)

To verify that genes in the subtractive libraries truly reflected their differential expression, qRT- PCR assays were developed for 4 of those that were down-regulated in infected birds (see later). Total RNA from marrow tissue of six birds randomly selected from each group (A and B) and each of the pools of RNA, used for SSH, were used.

Real-time PCR amplifications were performed in 25 μL reaction volume consisting of 2SYBR Green Mix (ABI, Foster City, USA) 12.5 μL, 10 μmol L^-1^ forward and reverse primers 1 μL, and ddH_2_O 9.5 μL, cDNA 1 μL, and performed with an ABI 7900 sequence detector (PE Applied Biosystems). Abundance of transcripts was normalized using the housekeeping gene 18S-rRNA (primer sequence shown in Table 1).

All real-time PCR reactions were performed in triplicate and standard curves were established in duplicate for each gene. Target genes and 18S rRNA were run in separate assays. Relative quantity ratios were obtained by dividing the relative quantity units of target genes by those of 18S rRNA. Mean values from triplicates were then used for statistical analyses. The specificity of the amplified fragments was routinely verified by melting curve analysis, using the Dissociation Curves software (PE Applied Biosystems). Relative quantification of gene amplification by real-time RT-PCR was performed using the cycle threshold (C_t_) values (Dawson *et al.*, 2004; Uthe *et al.*, 2006). Fold change in expression of the target gene is presented as log_2_ of the difference between averaged C_t_ values for control and infected-group chickens.

###  Statistical analysis

Data from real-time RT-PCR (C_t_ values) were analyzed using the unpaired t test from GraphPad InStat software (GraphPad Software Inc., San Diego, CA). Results were considered to be significant when p < 0.05.

## Results

###  Suppression subtractive hybridization (SSH) to identify differentially expressed genes

Two libraries of genes that were differentially expressed (up- and down-regulated) by ALV-J infection were constructed by SSH. Subtractions were validated when incapable of amplifying G3PDH from either subtracted SSH-cDNAs on using 18 to 33 cycles, although this was readily detected with as few as 18 cycles from non-subtracted cDNAs (data not shown).

Differential cDNA screening by Reverse Northern Blot hybridization was performed on 496 forward-subtracted cDNA clones and 384 reverse-subtracted cDNA clones, revealing 64 up-regulated and 40 down-regulated cDNA clones. In both libraries, several genes were represented by > 1 clone, indicating the likelihood that all cDNAs of interest had been screened. The mean size of cDNA was 312 bp and 324 bp in forward and reverse subtracted libraries, probably a result of the required restriction-digestion step in the SSH methodology used here. Of the 104 clones sequenced, 28 were duplicates, 17 sequences corresponded to genes of unknown function, and 59 discrete sequences were of known genes. The cellular roles of the differentially expressed genes involve the following functions: translation, transcription, immunity, cell metabolism, vesicular transport, signal transduction, cell cycle regulation, apoptosis and membrane proteins. Of special interest in this study; 22 genes (8 up-regulated and 14 down-regulated) were related to immune function, as shown in Tables 2  and 3.

###  Validation of differential expression by qRT-PCR

Four down-regulated genes from the SSH procedure, with potential roles in the hosts' immune and inflammatory response to ALV infection, were selected for confirmation of differential expression. Primers were designed for adult alpha D globin, gallus hemoglobin delta, ferritin H chain protein and carbonic anhydrase II genes from the sequences, and screened for specificity using BLAST. The results from qRT-PCR are presented in Table 4. As expected, all the 4 genes were expressed at significantly lower levels in ALV-J infected (Group A) than in non-infected (Group B) birds.

## Discussion

SSH is a gene expression profiling method specifically designed for comparing gene expression at various developmental stages or in altered physiological conditions (Diatchenko *et al.*, 1996). In the present study, use of SSH and Reverse Northern blot methods lead to identifying 59 partial gene sequences encoding proteins involved in major physiological functions. 22 major genes related immune function were identified, reflecting profound modifications of functional pathways: gene products involved in iron metabolism, those of the hemoglobin and globin family, TPT1 (a tumor protein) and MHC gene products. Altered expression of 4 of these identified genes, confirmed by RT-PCR, validated the SSH procedure.

###  Transferrin and ferritin gene

Hemoglobin, transferrin and ferritin play roles in iron metabolism. Body iron, most prominently represented in hemoglobin and ferritin, is conserved and recycled for further use (Aisen and Listowsky, 1980). Transferrin, providing an exchangeable pool of circulating plasma iron, is the most important physiological source of iron for both erythropoiesis (Ponka and Schulman, 1993), as well as the liver, spleen and bone marrow. Inadequate provision of transferrin impairs hemoglobin production, thus leading to anemia. Many conditions, including infection and malignancy, can depress transferrin levels (Sabbatini, 2000). Oshtrakh *et al.* (2006) showed that the ferritin-like iron in liver and spleen from chickens with lymphoid leukemia, measured by Mössbauer spectroscopy, was significantly lower than that in normal chicken tissues.

It is well known that rapidly growing cells, especially malignant cells, require more iron for their growth and metabolism than do resting cells (Yang *et al.*, 2001). Mechanisms for the regulation of iron related gene expression, such as transferrin, transferrin receptor and ferritin, have been intensively investigated in human diseases (Ward *et al.*, 1984; Nakamaki and Kawabata, 2004; Shackelford *et al.*, 2006). Kollia *et al.* (2003) demonstrated that iron import proteins might complement one another in acute myeloid leukemia cells. Yang *et al.* (2001) showed that in tumor tissue from 42 breast cancer patients, expression in both the transferrin receptor and ferritin H-chain was significantly correlated, and that the abundance of ferritin H- chain transcripts was directly related to the status of axillary lymph nodes, the presence of metastasis disease and the clinical stage.

The clinical significance of gene expression on transferrin receptor and ferritin in chicken tumors caused by ALV, including LL (lymphoid leukosis) and ML, has, as yet, not been reported. In the present study, both transferrin and ferritin (H-chain) were identified as down- regulated genes from reverse subtracted libraries, the latter as down- regulated in ALV-J infected birds being validated by real-time PCR.

###  Hemoglobin and globin genes

Hemoglobin, the oxygen-carrying pigment and predominant protein in red blood cells, occurs in 7 forms in chickens, with HbA and HbD being the main types in adults (Landes *et al.*, 1980). Each hemoglobin molecule consists of four heme moieties surrounding the protein globin. Chickens possess four β-globin and three α-globin genes. α^A^_2_β_2_ and α^D^_2_β_2_ assemble into HbA and HbD hemoglobin respectively, Landes *et al.*, 1980.

Chicken hematopoiesis is characterized by developmentally regulated alterations in globin gene expression as the site of blood formation changes (Neinhuis and Maniatis, 1987). In this study, the β-globin gene, α^A^ and α^D^ globin were down-regulated genes, whereas another chicken alpha-globin (neither alpha-A or alpha-D), and an alpha-globin that is abundantly expressed during hemolytic anemia, were up-regulated. Unsurprisingly, HbA1, HbA2 and HbD gene expression was less in ALV-J infected birds, although until now, little has come to light on the molecular relationship between hemoglobin synthesis and ALV infection. Up-regulation of the abundantly expressed alpha-globin in hemolytic anemia would be consistent with birds infected by ALV-J being anemic.

These results, taken together with the changes in expression of transferrin and ferritin genes suggest that ALV-J infection may first induce changes in iron metabolism, thereby causing changes in expression of those genes more directly involved in the synthesis of hemoglobin.

###  MHC genes

The B complex of the chicken major histocompatibility complex (MHC), which is known as the antigen-presenting structure, plays an important role in disease resistance and susceptibility to numerous pathogens, including both the Marek virus (Longenecker, 1977; Markowski-Grimsrud and Schat, 2002) and the Rous sarcoma virus (Bacon *et.al*.1981; Taylor, 2004). The relationship between avian leukosis virus and the B complex has already been studied. In three separate experiments, Bacon *et al.* (1981) infected chicks of different B complex haplotypes, with a standard inoculum from each of three tumor viruses (Rous sarcoma virus, Marek's disease, lymphoid leukosis). The B2 haplotype conveyed greater resistance than B5 to tumors caused by all the three viruses. This implies that certain gene(s) in this B-haplotype may determine a general ability to resist tumor formation or cause tumor regression. Yoo and Sheldon (1992) showed that the three haplotypes (B8a, B9a and B21) were similar, when evaluating fowl susceptibility to congenital infection by avian leukosis virus in hatching eggs, but differed in susceptibility to post-hatching infection from other infected birds. Mays *et al.* (2005) compared the responses of white leghorn chickens, of various B haplotypes, to ALV-J infection at hatching, and suggested that immune response to ALV came about through their influence.

In the present study, MHC B-G antigens turned out to be up-regulated genes identified from forward subtracted libraries, thereby implying that these may be of importance in facilitating ALV-J clearance by attracting immune cells to inflammation during an acute infection. Although the relationship between MHC and ALV-J infection have previously been shown at the DNA level, based on the gene expression results herein, we propose that MHC genes (or more specifically the MHC B-G gene) are those potential candidate genes most likely to be involved in the process of ALV-J infection and/or tumorigenesis.

In summary, this study was undertaken to investigate the transcriptional response to naturally occurring infection by ALV-J in 33 week-old chickens. In A-group chickens, test results were positive for the ALV-J virus, a type-specific antigen and the presence of an antibody, thereby indicating chronic infection. SSH methods identified and RT-PCR confirmed up- and down-regulation of those chicken genes most likely to be involved in the response to chronic infection. This is yet an initial attempt towards a genome-wide gene expression analysis in bone marrow tissue and toward understanding the infection process, tumorigenesis and the induced immune reactions in this important disease condition. Our intension is to continue investigating the physiological/ pathological functions of these gene products, their possible roles in ALV infection and tumor development, and their potential utility as markers for aiding in the selection of birds with enhanced resistance to the virus itself.

## Figures and Tables

**Table 1:** PCR primers of down-regulated genes for detecting levels of mRNA expression.

Clone I.D.	GenBank accession n.	Identified gene	PCR primers	RT-PCR amplicon (bp)
1-122	NM_001004375	Gallus adult alpha D globin	5-TCCCACCAGGAGGAGTTTG-3 3-TTCTTGCACCTGTTGGAGTCG-3	77
1-148	NM_205489	Gallus hemoglobin, delta	3-TTCTTGCACCTGTTGGAGTCG-3 3-TTCTTGCACCTGTTGGAGTCG-3	147
1-149	Y14698|	Gallus mRNA for ferritin H chain protein	5-GGGTGGACGCATCTTCTT-3 3-TGG TTG GAC GCC TTC TAC -5	247
1-44	X17378	Carbonic anhydrase II, exon 7	5-CTAAAGAGCAGGGAAGTCAG-3 5-CAGTCGCAGTAAGTCATCAA-3	288
	AF173612	*18S rRNA*	5-TAGATAACCTCGAGCCGATCGCA-3 5-GACTTGCCCTCCAATGGATCCTC-3	300
	AF047874	*GAPDH*	5-TCACAAGTTTCCCGTTCTCA-3 5-GGAACACTATAAAGGCGAGAT-3	206

**Table 2:** Identities of selected cDNA clones present in forward subtracted libraries.

Clone I.D.	BLASTn submission (bp)	Gene description
845;852	330	Chicken messenger RNA for an alpha-globin abundantly expressed during hemolytic anemia
84	379	Messenger RNA for a new chicken alpha-globin (neither alpha-A or alpha-D)
543;440	180	Chicken MHC B-G antigen mRNA, partial cds, clone G6
88;766	320	Natural killer cell granular protease (RNK-Met-1)
640	233	Chicken ubiquitin mRNA from heat shocked embryo cells
805	225	Chicken insulinoma (rig) mRNA, complete cds
901	163	Similar to nuclear zinc finger protein RAP80
834	286	Human DNA sequence from clone RP4-680D5 on chromosome 1p36.13-36.31

**Table 3:** Identities of selected cDNA clones present in reverse subtracted libraries.

Clone I.D	BLASTn submission (bp)	Gene description
1-122;1-51;1-252	426	Gallus adult alpha D globin (loc416651), mRNA
1-229; 1-97	226	Gallus adult alpha A globin, mRNA
1-25	184	Gallus adult Beta globin, mRNA
1-179	247	Gallus hemoglobin, alpha 1(HBA1), mRNA
1-129	307	Gallus hemoglobin, alpha 2 (HBA2), mRNA
1-148; 1-191	312	Gallus hemoglobin, delta (HBD) , mRNA
1-280	420	Gallus gallus transferrin (TF)
1-149	343	Gallus mRNA for ferritin H chain protein
1-104	320	Gallus ferritin heavy polypeptide 1 (FTH1)
1-264	366	Gallus bHLH transcription factor beta3
1-139	376	H/K Gallus gallus ATPase, H+/K+ transporting, alpha polypeptide
1-44	205	Chicken mRNA for carbonic anhydrase II, exon 7
1-254	202	Gallus gallus tumor protein, translationally-controlled 1 (TPT1)
1-256	302	Gallus similar to receptor protein tyrosine phosphatase LAR

**Table 4:** Gene expression from chicken bone marrow during ALV-J infection.

Clone I.D.	Identified gene	Infected group*	Non-infected group*	p-value
1-122	adult alpha D globin	0.30 ± 0.07	35.93 ± 2.02	p < 0.001
1-148	gallus hemoglobin, delta	3.64 ± 1.62	50.10 ± 5.59	p < 0.001
1-149	ferritin H chain protein	73.51 ± 16.79	521.14 ± 14.16	p < 0.001
1-44	carbonic anhydrase II	40.77 ± 23.15	84.01 ± 10.11	p < 0.001

*Data expressed as mean ± SD.
